# Detection of extended-spectrum beta-lactamase-producing *E. coli* and *Klebsiella* spp. in effluents of different hospitals sewage in Biratnagar, Nepal

**DOI:** 10.1186/s13104-019-4689-y

**Published:** 2019-10-04

**Authors:** Sanjay Mahato, Ajay Mahato, Elina Pokharel, Ankita Tamrakar

**Affiliations:** 1 Aasra Research and Education Academy Counsel, Janapriya Tole, Biratnagar-6, Nepal; 2 Department of Microbiology, Mahendra Morang Adarsha Multiple Campus, Tribhuvan University, Biratnagar, Nepal; 3 Department of Orthopaedics, Birat Medical College and Teaching Hospital, Biratnagar, Nepal

**Keywords:** Hospital sewage, *Klebsiella*, *E. coli*, MDR, ESBL, Water

## Abstract

**Objective:**

This study was aimed to determine prevalence and resistance pattern like multidrug resistant (MDR) or ESBL nature of *E. coli* and *Klebsiella* spp. from various sewage drain samples with an idea to deliver baseline information that could be utilized for defining guidelines for the treatment of hospital sewages.

**Results:**

Of 10 sewage samples analyzed, 7 (70%) contained *E. coli* while 6 (60%) contained *Klebsiella.* Except one sample, all positive samples contained both *E. coli* and *Klebsiella* spp. *E. coli* isolates were resistant to ampicillin, amoxicillin, cefoxitin, cefuroxime, and cefpodoxime; while 85.7% were resistant to amoxicillin/clavulanate, ceftazidime, cefotaxime and ceftriaxone. 71.4%, 57.1%, 42.9%, and 28.6% were resistant to aztreonam, trimethoprim/sulfamethoxazole, nitrofurantoin, and gentamicin. Most were sensitive to chloramphenicol, ofloxacin, ciprofloxacin, and azithromycin. 85.7% and 57.1% of *E. coli* were MDR and ESBL isolates, respectively. *Klebsiella* were resistant to ampicillin, amoxicillin, and amoxicillin/clavulanate. 83.4% of *Klebsiella* were resistant to cefoxitin. 66.7% of strains were resistant to cefuroxime, ceftazidime, cefotaxime, ceftriaxone, and cefpodoxime. *Klebsiella* showed 50% resistant to aztreonam and trimethoprim/sulfamethoxazole, while 33.3% were resistant to chloramphenicol, nitrofurantoin, ofloxacin, and ciprofloxacin. *Klebsiella* were sensitive to azithromycin and gentamicin. 66.7% and 33.3% of *Klebsiella* were MDR and ESBL isolates, respectively.

## Introduction

Sewage designates raw sewage, sewage sludge or septic tank waste containing about 95.5% water and 0.1% to 0.5% organic and inorganic materials. Hospital wastewaters are generated in different sections of hospital like surgery units, ICU, laboratories, patient wards, clinical wards, laundries and possess a quite variable compositions depending on the activities involved [1]. A variety of microorganisms are present in water for examples bacteria, fungi, protozoa etc. Bacteria like *Shigella*, *E. coli*, *Klebsiella*, *Vibrio*, *Salmonella*, etc. are found in sewage drain water [2]. Coliforms are mainly from family *Enterobacteriaceae* that are aerobic or facultative aerobic, gram negative, non-spore forming enteric bacilli and basically found in human colon which are introduced into environment by human feces [3, 4].

Antibiotics are fractionally metabolized by patients and are then ejected into the hospital sewage. Along with excreta, they pass through sewage system and end up in the environment, mainly in the water area [5]. Hospital sewage discharge a variety of multi resistant bacteria and substances like antimicrobial, pharmaceutical, disinfectants, heavy metals, radioisotopes, and drugs not metabolized by patients [6].

Multiple drug resistance (MDR) for Gram negative and Gram-positive bacteria means “resistant to three or more antimicrobial classes.” Extended-spectrum β-lactamases (ESBL) are enzymes that impart resistant to extended-spectrum (third generation) cephalosporins (e.g. ceftazidime, cefotaxime and ceftriaxone) and monobactams (e.g. aztreonam). The most common ESBL producing bacteria are few strains of *E. coli* and *Klebsiella pneumoniae* [7, 8].

The main aim of this study was to determine prevalence and resistance pattern like MDR or ESBL nature of *E. coli* and *Klebsiella pneumoniae* from various sewage drain samples since they can cause serious public health problem. This study could deliver baseline information that could be utilized for defining guidelines for the treatment of hospital sewages.

## Main text

### Methods

#### Sample collection method and characteristics

During March to October 2018, a total of 10 sewages samples were collected aseptically from different hospitals of Biratnagar city (Table 1). For this, the sample was collected nearby the center of the flow channel, at approximately 10–15 cm depth from the water surface, where the turbulence was at maximum and the possibility of settling was minimized. Skimming the water surface or dragging the bottle was avoided. The sewage water was first mixed and then 500 ml sample was taken in the sterile high-density polyethylene (HDPE) bottle aseptically. Each sample bottle was properly labelled with date, code number and time with the help of the marker.

**Table 1:** Sample collection detail of hospitals from Biratnagar, Nepal

Sample	Date	Location	Code	*Klebsiella*	*E. coli*	Code
1	0125	Saptakoshi Hospital	S1LP	+	+	S1P
2	0125	Saptakoshi Hospital	S2LP	+	+	S2G
3	0131	Tulasa Mother and Child Hospital	S3LP	+	+	S3G
4	0131	Max International Hospital	S4	−	−	S4
5	0204	Lifeguard Hospital	S5LP	+	+	S5G
6	0406	Morang Sahakari Hospital	S6	−	−	S6
7	0406	Morang Sahakari Hospital	S7	−	−	S7
8	0408	Koshi Zonal Hospital	S8LP	+	+	S8G
9	0408	Koshi Zonal Hospital	S9	−	+	S9LP
10	0408	Koshi Zonal Hospital	S10LP	+	+	S10LP

#### Sample transportation and processing

Samples collected were placed on 4 °C ice box to inhibit the growth of microorganisms and were immediately transported (within 2 h) to microbiology research lab for the analysis. Distilled water was used as control during analysis.

#### Isolation and identification of *E. coli* and *Klebsiella* spp.

The samples collected from the hospital wastewater were serially diluted in 0.85% saline water and dilution 10^−2^ and 10^−3^ were inoculated onto Eosin Methylene Blue (EMB) Agar and MacConkey Agar for *Escherichia coli* and *Klebsiella* spp. by spread plate method and were incubated aerobically at 37 °C for 24–48 h. After incubation, colonies were picked on their colony morphology like colonial appearance, size, elevation, color, margin, and opacity. All the selected colonies were, then, sub-cultured on nutrient agar plate to obtain pure culture for the microscopic and biochemical identification. TSI (triple sugar iron), SIM (sulfate/indole/motility), Methyl Red test, Voges–Proskauer test, citrate agar, catalase test, oxidase test, and urea hydrolysis test were performed to identify the organisms [9].

#### Antimicrobial susceptibility tests

The identified isolates of *Escherichia coli* and *Klebsiella* spp. were submitted to antimicrobial susceptibility testing according to the guidelines of the Clinical and Laboratory Standards Institute [10]. The isolates were inoculated onto Mueller‐Hinton agar medium using turbidity of 0.5 McFarland standard. The following antimicrobial disk (Himedia, Mumbai, India) were used: Ampicillin (AMP) (10 μg), Amoxicillin (AMX) (10 μg), Amoxicillin/clavulanate (AMC) (20/10 μg), Cefoxitin (CX) (30 μg), Ceftazidime (CAZ) (30 μg), Ceftriaxone (CTR) (30 μg), Cefpodoxime (CPD) (10 μg), Cefuroxime (CXM) (30 μg), Aztreonam (AT) (30 μg), Chloramphenicol (C) (30 μg), Azithromycin (AZM) (15 μg), Gentamicin (GEN) (30 μg), Ciprofloxacin (CIP) (5 μg), Ofloxacin (OF) (5 μg), Nitrofurantoin (NIT) (300 μg), and Trimethoprim/sulfamethoxazole (COT) (1.25/23.75 μg). The swabbed MHA plates with the discs were incubated at 37 °C for 24 h. Zone of inhibition was measured and interpreted using the standard chart [10]. Due to unavailability of ATCC culture, sensitive *E. coli* and *Klebsiella pneumoniae* strains with established antibiogram were used as control.

#### Criterion for multidrug resistance

Isolates which demonstrated the resistance to at least one agent in three or more classes of the drug were defined as multidrug resistant (MDR) [10, 11].

#### ESBL detection

Isolates exhibiting a zone of inhibition of growth for ceftazidime and ceftriaxone ≤ 22 mm and ≤ 25 mm, respectively, were submitted to the combined disc test to check for ESBL‐producing strains [12]. The combined disc methodology used to detect ESBL‐producing *E. coli* and *Klebsiella* spp. was performed as per CLSI [10]. The antimicrobials used were ceftazidime (30 μg) and ceftazidime/clavulanic acid (30/10 μg), and cefotaxime (30 μg) and cefotaxime/clavulanic acid (30/10 μg). Results were interpreted as per the criteria established by the CLSI [10]. An increase of 5 mm in a zone of inhibition of growth for combined drugs to ceftazidime or cefotaxime were confirmatory for ESBL‐producing strains [10, 12].

#### Multiple antibiotic resistance (MAR) index

MAR index is the number of antibiotics to which test isolate displayed resistance divided by the total number of antibiotics to which the test organism has been evaluated for sensitivity. MAR index for each isolate was calculated as per the guidelines of Krumperman [13].

#### Data analysis

The data were statistically analyzed using Statistical Package for Social Sciences (SPSS v21) software package. Chi square test at p-value < 0.05 was considered statistically significant.

### Results

Out of 10 samples analyzed, 7 (70%) contained *E. coli* while 6 (60%) contained *Klebsiella.* Except one sample, all positive samples contained both *E. coli* and *Klebsiella* spp.

Colonies with green metallic sheen on EMB agar on further analysis were confirmed to be *E. coli.* Microscopic examinations revealed them to be gram negative non-capsulated bacilli (1SH, 2SH, 3TMC, 5LG, 8KZ, 9KZ, 10KZ). All the 7 isolates were motile, non-hydrogen sulfide producers; VP, citrate, oxidase negative while was indole, methyl red, catalase, urease, TSI (acid/acid with gas) positive. Pink colored, highly mucoid colonies in EMB Agar on further examinations were found to be *Klebsiella pneumoniae*. Microscopic examinations revealed them to be gram negative capsulated bacilli (1SH, 2SH, 3TMC, 5LG, 8KZ, 10KZ). All the 6 isolates were non-motile, non-hydrogen sulfide producers; indole, MR, oxidase negative while was VP, citrate, catalase, urease, TSI (acid/acid with gas) positive.

Out of 7 samples (n = 7) of *E. coli*, all the isolates were resistant to ampicillin, amoxicillin, cefoxitin, cefuroxime, and cefpodoxime. 85.7% of *E. coli* were resistant to amoxicillin/clavulanate and cephalosporins like ceftazidime, cefotaxime and ceftriaxone. The resistance shown by *E. coli* to aztreonam, Trimethoprim/sulfamethoxazole, nitrofurantoin, and gentamicin were 71.4%, 57.1%, 42.9%, and 28.6%, respectively (Table 2). 14.3% of strains were resistant to chloramphenicol and fluoroquinolones like ofloxacin, ciprofloxacin. All the strains were sensitive to azithromycin. Out of 7 isolates, 6 (85.7%) of *E. coli* (1SH, 2SH, 3TMC, 5LG, 9KZ, 10KZ) were multidrug resistant (MDR) bacteria. Notably 4 isolates (57.1%) of *E. coli* (1SH, 2SH, 9KZ, 10KZ) were confirmed as ESBL producing isolates.

**Table 2:** Antibiotic susceptibility pattern of *Klebsiella* and *E. coli* in percentage

Antibiotics	Resistance percentage (%)	
	*Klebsiella*	*E. coli*
Ceftazidime	66.7	85.7
Cefotaxime	66.7	85.7
Cefoxitin	66.7	57.1
Ceftriaxone	66.7	85.7
Cefpodoxime	66.7	100
Cefuroxime	66.7	85.7
Chloramphenicol	50	14.3
Gentamicin	0	28.6
Amoxicillin/clavulanate	100	14.3
Ampicillin	100	100
Amoxicillin	100	100
Aztreonam	50	71.4
Ciprofloxacin	33.3	14.3
Ofloxacin	33.3	14.3
Azithromycin	16.7	0
Nitrofurantoin	66.7	0
Trimethoprim/sulfamethoxazole	50	57.1

*Klebsiella pneumoniae* were resistant to ampicillin, amoxicillin, and amoxicillin/clavulanate. 83.4% of *Klebsiella* were resistant to cefoxitin; while 66.7% were resistant to cefuroxime, ceftazidime, cefotaxime, ceftriaxone, and cefpodoxime (Table 2). *Klebsiella* showed 50% resistant to aztreonam and Trimethoprim/sulfamethoxazole. 33.3% of strains were resistant to chloramphenicol, nitrofurantoin, ofloxacin, and ciprofloxacin. Only 16.7% of strains were resistant to azithromycin while were fully sensitive to gentamicin. Out of 6 *Klebsiella*, only 4 (66.7%) (1SH, 2SH, 3TMC, 8KZ) were MDR. Two isolates (33.3%) of *Klebsiella* (2SH, 8KZ) were confirmed to be ESBL producing isolates.

**Fig. 1:**
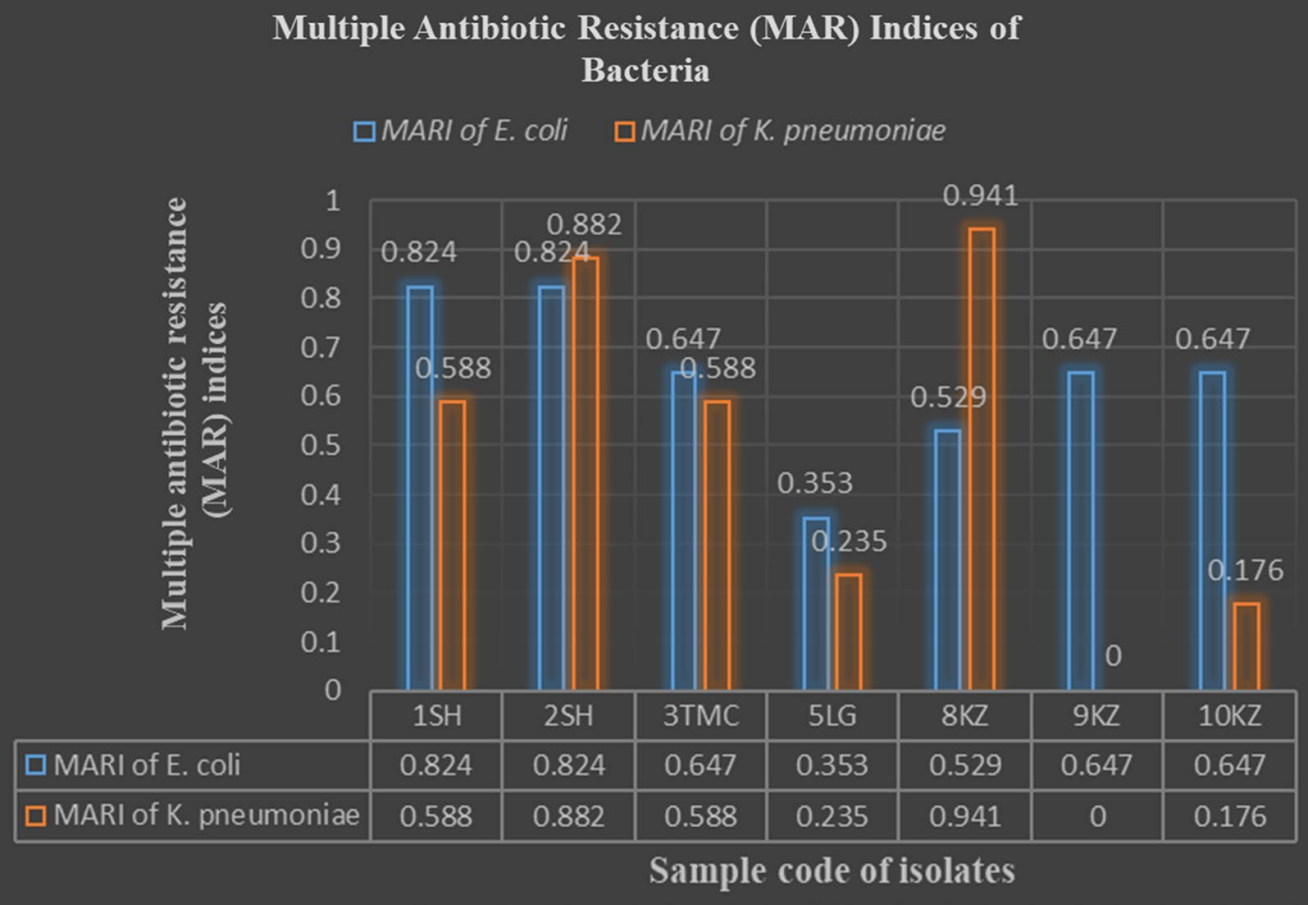
Multiple antibiotic resistance (MAR) indices of bacteria. MAR indices of bacteria revealed that none of *E. coli* and *Klebsiella* were susceptible or resistant to all the seventeen tested drugs

Multiple antibiotic resistance (MAR) indices of bacteria revealed that none of *E. coli* and *Klebsiella* were susceptible or resistant to all the seventeen tested drugs (Fig. 1). Of all 7 *E. coli*, 1 (14.3%) was resistant to 6 drugs (MARI = 0.353), 1 (14.3%) was resistant to 9 drugs (MARI = 0.529), 3 (42.9%) were resistant to 11 drugs (MARI = 0.647), and 2 (28.6%) was to 14 drugs (MARI = 0.824). Of all 6 *Klebsiella*, 1 (16.7%) was resistant to 3 drugs (MARI = 0.176), 1 (16.7%) was resistant to 4 drugs (MARI = 0.235), 2 (33.3%) were resistant to 10 drugs (MARI = 0.588), 1 (16.7%) was resistant to 15 drugs (MARI = 0.882), and 1 (16.7%) was to 16 drugs (MARI = 0.941).

There is no significant relationship between the type of bacterial strains (like *E. coli* and *Klebsiella*) and their response to the antibiotics at df = 1, p = 0.05.

### Discussion

The main aim of this study was to determine prevalence and resistance pattern like MDR or ESBL nature of *E. coli* and *Klebsiella pneumoniae* from various sewage drain samples. Presence of 70% of *E. coli* and 60% of *Klebsiella* in sewage may have direct link with the human feces in many cases [14]. Sewage mass is liquid mass containing excessive amount of organic matter which acts as a nutrient medium for all the bacteria [15]. Excessive number of MDR and ESBL *E. coli* and *Klebsiella* show that drainage system of Biratnagar hospitals is highly infectious and life threatening if contaminated with water and food [16].

The result of *E. coli* showing 100% resistance to ampicillin, amoxicillin, cefoxitin, cefuroxime, and cefpodoxime was higher than Belachew et al. [17] showing 91.3% resistance to ampicillin, 70% resistance to cefuroxime and ceftriaxone, Cefpodoxime (74%), amoxicillin/clavulanate (52%), cefoxitin (43%), ceftazidime (65%). In this study, 85.7% of *E. coli* were resistant to amoxicillin/clavulanate, ceftazidime, cefotaxime and ceftriaxone. Resistance to nitrofurantoin was similar in Belachew et al. [17]. Resistance to aztreonam and chloramphenicol were higher than the findings of Florica et al. [18]. On the contrary, resistance to trimethoprim/sulfamethoxazole (57.1%), gentamicin (28.6%) and ciprofloxacin (14.3%) in our study was lower than Belachew et al. [17] showing 67%, 43%, and 52%, respectively. It has been observed that none of the hospitals in Biratnagar have waste treatment system as a result, 85.7% of *E*. *coli* species had multi-drug resistance, which is, higher compared to previously reported results in Ethiopia (78%) [17] and Romania (60.34%) [18]. Such a high resistance rate may be a result of poor waste management practice, lack of treatment plants for healthcare institutions and poor antimicrobial usage in Biratnagar.

*Klebsiella pneumoniae* showed 100% resistance to ampicillin and amoxicillin/clavulanate which was higher than the study of Ethiopia (94%) [17] and Romania (70.7%) [18]. Resistance to penicillin antibiotics like ampicillin has become very common in the world and our finding is in line with this evidence. Resistance of 66.7% for ceftazidime, cefotaxime, and ceftriaxone was higher than the findings of Romania [18] as 8.6%, 17.2% and 13.8%, respectively. Resistance shown by Trimethoprim/sulfamethoxazole (50%), chloramphenicol (33.3%) and ciprofloxacin (33.3%) were much higher than found in Romania as 22.4%, 5.2% and 6.9%. Similarly, the resistance to cefoxitin, cefuroxime, cefpodoxime, and nitrofurantoin in this study was found to be much higher than other studies [17]. A high rate of MDR (66.7%) was observed for *Klebsiella* spp. which was higher than results reported in Ethiopia (40.5%) [17], Romania (33%) [18] and Mexico (50%) [19]. However, MDR rate of the current finding was lower than previously reported results in Brazil (77.5%) [20]. Such variation may be due to the difference in antimicrobial use and availability of waste treatment system in hospital sewage [17].

### Conclusion

This study builds the importance to enquire the involvement of hospital liquid waste discharge in the development and distribution of antibiotics resistance in the environment. There is rise in resistant bacteria like *E. coli* and *Klebsiella* in hospital wastewater. The government must implement some rules and laws for proper treatment of hospital wastewater before entry to main municipal wastewater. Sewage treatment plant must be established in hospital for their effluents and sludge coming from the hospital’s units.

## Limitations

The standard strain *E. coli* (ATCC 25922) and *K. pneumoniae* (ATCC 13883) could not be used.

## Data Availability

All the required data and material of research is given in the manuscript.

## Abbreviations

MDR: multiple drug resistance
ESBL: extended-spectrum β-lactamases
ICU: intensive care unit
HDPE: high density polyethylene
EMB: Eosin Methylene Blue
TSI: triple sugar iron
SIM: sulfate/indole/motility
CLSI: Clinical and Laboratory Standards Institute
MHA: Mueller‐Hinton agar
AMP: ampicillin
AMX: amoxicillin
AMC: amoxicillin/clavulanate
CX: cefoxitin
CAZ: ceftazidime
CTR: ceftriaxone
CPD: cefpodoxime
CXM: cefuroxime
AT: aztreonam
C: chloramphenicol
AZM: azithromycin
GEN: gentamicin
CIP: ciprofloxacin
OF: ofloxacin
NIT: nitrofurantoin
COT: trimethoprim/sulfamethoxazole
μg: microgram
ml: milliliter
MAR: multiple antibiotic resistance
MAR: multiple antibiotic resistance indices
WHO: World Health Organization
